# The Influence of the Playing Surface on Workload Response in Spanish Professional Male Soccer Players

**DOI:** 10.3390/s24144506

**Published:** 2024-07-12

**Authors:** José C. Ponce-Bordón, Jorge Polo-Tejada, Borja Sanabria-Pino, Ana Rubio-Morales, Tomás García-Calvo, David Lobo-Triviño

**Affiliations:** Faculty of Sport Sciences, University of Extremadura, Avda. De la Universidad, S/N, 10003 Cáceres, Spain; joponceb@unex.es (J.C.P.-B.); jpolotej99@gmail.com (J.P.-T.); bsanabriapino@gmail.com (B.S.-P.); rubiomorales@unex.es (A.R.-M.); davidlt@unex.es (D.L.-T.)

**Keywords:** assessment, football, mental load, monitoring, pitch types, training load

## Abstract

This study aimed to quantify the influence of the playing surface on workload-related variables (i.e., external load, Rate of perceived exertion (RPE), and mental load) in training sessions with a Spanish professional soccer team. Twenty professional male players from the same soccer team were involved. A total of thirty training sessions related to the preseason period were included. All the players completed training sessions on three playing surfaces: natural turf of poor quality, natural turf of high quality, and third-generation artificial turf. Monitoring during sessions involved assessing internal load (i.e., RPE and mental load) via self-reported questionnaires, and external load using Global Positioning System devices. Linear mixed models showed that RPE was significantly higher on natural turf of high quality than on natural turf of poor quality (*p* < 0.001). Total distance, relative total distance, the number of accelerations, decelerations, and high metabolic load distance were significantly lower on third-generation artificial turf compared to natural turf of poor quality (*p* < 0.001) and high quality (*p* < 0.001). In addition, high-speed running, sprint running distances, and the number of sprints reached higher values on third-generation artificial turf compared to the other two playing surfaces. These findings highlight the need for coaches to consider the type of training surface in soccer to optimize training load planning and prevent injuries.

## 1. Introduction

Soccer is by far the most popular sport among youth in the world. The international federation of association football (FIFA) has reported that in 2006 there were 270 million people practicing soccer, almost 4% of the world’s population [1]. In particular, the federated practice of soccer has experienced extraordinary growth in Spanish society in the past few decades [2]. The increase in the number of licenses registered by the Spanish Royal Federation of Football made clear this growth: in 2011, the number of licenses had grown to 19 times that of 50 years earlier. In addition, the total impact of Spanish soccer on the economy is approximately 1.7% of the overall GNP, and it provides employment to almost 66,000 people [2]. Consequently, a large number of soccer clubs have become more professional in their daily routine, with better resources to increase the soccer performance of players [3].

Soccer is an intermittent team sport, where high-intensity actions have great importance [4,5]. Specifically, an increase in high-intensity actions during matches, such as jumping, sprinting, or changes in direction, has been observed in the last few years [6]. In addition, success in soccer is closely related to high-intensity actions; therefore, players should have adequate preparation to achieve optimal performance [7]. However, it should be considered that high-intensity variables are influenced by contextual variables, such as match outcome and match location [8]. For example, players may experience different training demands during the week after losing a match compared to the week after winning [9]. The objective of the monitoring process is to examine how soccer players cope with the training load, and how the players are adapting to the training stimulus [10]. Therefore, it seems essential to know the weekly demands of soccer players through load quantification strategies in order to optimize performance and reduce the risk of injury [11].

For this purpose, one of the most relevant strategies in recent years to quantify external loads has been the inclusion of Global Positioning System (GPS) devices in professional and semi-professional soccer clubs [12]. These instruments help to optimize rehabilitation programs [13], improve performance [14,15], and prevent injuries [16]. However, soccer is physically as well as mentally demanding due to the continuous perceptual–cognitive and emotional stimuli [17,18]. Highly mentally demanding contexts (i.e., with a high perceived cognitive and/or emotional load) can induce a state of mental fatigue in soccer players [19] and, consequently, cause impairments in performance. For example, Sun et al. [20] reported that mental fatigue negatively influences the technical performance of soccer players. At the physical level, mental fatigue decreases exercise tolerance through increases in the ratio of perceived exertion (RPE) [21,22]. Mental fatigue also negatively affects tactical and cognitive performance, resulting in poorer space synchronization between players [22,23]. Finally, according to Kunrath et al. [24], mental fatigue may also cause an increased risk of muscle injury in soccer players. Although the number of research studies that examine the weekly workload is increasing, new studies that jointly analyze external and internal load variables are needed to obtain more information about the condition of the players during the microcycle.

Additionally, training load analysis should also consider that there are different variables or environmental factors, like playing surfaces, which can influence the workload of soccer players [25]. Soccer has been traditionally practiced on natural grass; however, the improvements in artificial turf systems have led to an increased acceptance of third-generation artificial surfaces in soccer clubs [26,27,28]. Consequently, the influence of playing surfaces on technical and physical components of soccer performance has been increasingly studied over the last few years [29]. For instance, according to Modric et al. [30], playing on artificial turf is more physically demanding for defensive and midfield players than playing on natural grass. In the same line, Brito et al. [31] found that the total distance and the very-high-intensity running covered by the players were significantly higher on artificial turf than on natural grass. Regarding technical actions, the total number of passes per team per game was higher on artificial turf than on natural grass [32]. Therefore, knowing the demands of training on different types of surfaces is essential to adapt the weekly workload to optimize the players’ preparation and reduce the risk of injury.

Accordingly, due to the importance of jointly quantifying mental and physical training loads for optimizing the performance of soccer players, and considering the possible influence of different playing surfaces on workload on soccer training sessions, this study aimed to analyze the effects of playing surface (i.e., natural turf of poor quality, natural turf of high quality, and third-generation artificial turf) on workload-related variables (i.e., mental load, RPE, and external load) in professional Spanish soccer players. Based on findings from previous studies, we hypothesized that: (i) mental load is higher on artificial turf than natural turf [32]; (ii) RPE values are lower on artificial turf than on natural turf [30]; (iii) finally, external load variables are higher on artificial turf than natural turf, specifically, total distance (TD) and high-speed running (HSR) distance [33].

## 2. Methods

### 2.1. Sample

A total of twenty professional male soccer players (M_age_ = 26.16 ± 5.32) were involved in the current study. All the athletes were recruited from a professional Spanish team (Third Division) competing in the national league. They performed five field-training sessions per week (80 min on average per session) over six weeks of the preseason period and played one competitive match per week. Although all the data were collected as a condition of employment, in which players are monitored daily over the competitive season, approval for the study from the club was also obtained. Before starting this research, the participants signed an informed consent form that explained the purposes and potential risks associated with participating in this research. The procedures of this study were approved by the local ethics committee (University of Extremadura; protocol number: 33/2024). All the data were treated according to the privacy, ethics, and protection policies of the American Psychological Association (2020).

### 2.2. Study Design

A retrospective, descriptive longitudinal design (i.e., no intervention during training) was conducted to analyze the differences in workload-related variables between different playing surfaces during training sessions. During the preseason period of the 2023/24 season (from July to August), a total of thirty training sessions were performed and analyzed. The soccer players who participated in the study were always the same. The goalkeepers participated in the training sessions but were excluded from the analysis due to their specific roles. Extra soccer balls were always available near the goalposts and on the side of the pitch for prompt replacement when the ball left the playing area. All the training sessions were performed between 9 and 11 am. To increase the ecological validity of our study, all the analyses were conducted on regulation soccer pitches. In addition, all the training sessions were preceded by a planned, standardized warm-up for 12 min that included 5 min of running activities and 5 min of exercises of articulation mobility with increasing intensity, with a recovery process of 2 min before the main task. Stretching exercises were not carried out during the warm-up. Additionally, a structured cool-down, which included static stretching exercises and foam rolling exercises, was implemented at the end of the training sessions to enhance recovery between training sessions [34].

The external, internal, and mental load were recorded by Global Positioning System (GPS) devices and self-reported questionnaires during training sessions in the preseason period. The playing surfaces were classified into three groups: natural turf of poor quality (Grass1; *n* = 168 observations, 13 sessions); natural turf of high quality (Grass2; *n* = 437 observations, 30 sessions); and third-generation artificial turf (3G; *n* = 195 observations, 13 sessions) comprising long synthetic grass with shock-absorbent rubber crumb infill between the grass fibers [35]. The surfaces were dry before the training sessions, with watering of the turfs occurring in the preceding hours [35].

### 2.3. Procedure and Variables

#### 2.3.1. External Load

The external load data were consistently monitored across the full study season during all the training sessions using an 18 Hz Global Positioning System (GPS) technology tracking system (Apex Pod, version 4.03, 50 g, 88 × 33 mm; STATSports; Northern Ireland, UK). These Apex devices were previously validated for tracking distance covered and peak speed in team sports [12]. All the devices were activated 20 min before data collection to allow the acquisition of satellite signals and to synchronize the GPS clock with the satellite’s atomic clock [36]. To avoid inter-unit error, each player wore the same device during the study period [37], although the present GPS system has been previously reported to show excellent inter-unit reliability [38]. After each session was completed, the GPS data were extracted using software (Apex, 10 Hz version 4.3.8, STATSports; Northern Ireland, UK), as software-derived data is a more simple and efficient way for practitioners to collect data in an applied environment, without differences reported between processing methods (software-derived to raw processed) [39]. The dwell time (minimum effort duration) was set at 1 s to detect high-intensity running and sprint distance efforts, in line with the manufacturer’s recommendations and default settings to maintain consistent data processing [38]. STATSports provided written permission to allow all the data to be used for research purposes. For further analysis, the following variables were considered: total distance covered by soccer players (TD) in meters; relative total distance covered by soccer players in meters per minute; medium-speed running distance (MSR, distance 18–21 km·h^−1^); high-speed running (HSR, distance > 21 km·h^−1^); very-high-speed running (VHSR, distance 21–24 km·h^−1^); sprinting speed running distance (Sprint, distance > 24 km·h^−1^); the number of sprints performed by soccer players (Sprints, number of physical efforts > 24 km·h^−1^); the number of total high accelerations (ACC > 3 m·s^−2^); total high decelerations (DEC > 3 m·s^−2^); and the high metabolic load distance (HMLD; this value corresponds to running at a constant speed of 5.5 m·s^−1^ or 19.8 km·h^−1^ on grass). All the variables are presented as the mean value per session.

#### 2.3.2. Internal Load

To examine the subjective feelings of mental load after each field-training session (i.e., 0–30 min after each session), an adaptation of the Questionnaire to quantify the Mental Load in Team Sports was used (Figure S1; [17]). From the four-item questionnaire, only two items: (i) rating of perceived exertion (RPE; How demanding would you quantify the physical effort of this session?) [40] and (ii) cognitive load (How demanding would you quantify the cognitive effort of this session?), were applied. This instrument uses a Likert scale format with a response range of 0 (no effort perceived) to 10 (maximum effort perceived) for each question. In this adaptation, the players rated only one soccer-related issue: mental effort. This questionnaire has demonstrated reliability (Cronbach’s α = 0.73, and ω = 0.75) and validity (factor structure for RPE = 0.81, and cognitive load = 0.72) for the assessment of mental load in team sports and has been previously applied for this purpose in soccer studies [17,41,42].

### 2.4. Statistical Analysis

All statistical analyses were performed using RStudio [43]. Firstly, we performed an unconditional null model using the MIXED procedure to calculate each outcome measure’s intraclass correlation coefficient (ICC). The ICC’s standard error and 95% confidence interval were also calculated. The marginal and conditional R^2^ values were also considered to analyze the model fits. 

Once the adequacy of the statistical procedure was determined using the ICC values, and considering the characteristics of the sample, organized hierarchically and in groups, and with a longitudinal structure, we considered that the best procedure to analyze the data was through linear mixed models (LMMs). LMMs have been demonstrated to cope with unbalanced and repeated-measure data [44,45]. For instance, workload variables in training sessions are grouped by players (i.e., each player has a record for every training session they have participated in, and each training session has observations of several players). Thus, the cross-classified multilevel models are suitable for data structures that are not purely hierarchical. Consequently, a general multilevel modeling strategy was applied where fixed and random effects in different steps were included as per Heck et al. [45].

Thus, LMMs were applied to examine the effects of playing surfaces on training load variables. First, a two-level hierarchy was modeled for the analysis. Workload variables (i.e., mental load, internal, and external load parameters) were included as dependent variables in the models, and playing surface (i.e., Grass1, Grass2, and 3G) were the independent variables included as fixed effects. The soccer player was considered as the random effect in the analysis. Values were represented as coefficients and standard error (Coeff ± SE). Statistical significance was set at *p* < 0.05. Finally, for the calculation of the effect size (ES) of the model, eta squared (η^2^) was calculated with the following ranges: 0.01: low; 0.06: middle; and 0.14: elevated; while to quantify the magnitude of difference for all pairwise comparisons, Cohen’s D was also performed and interpreted as follows: 0.00–0.19: trivial; 0.20–0.59: small; 0.60–1.19: moderate; 1.20–1.99: large; and ≥2.00: very large [46].

## 3. Results

Table 1 and Table 2 show the marginal and conditional R^2^ values, the ICC values and their 95% confidence intervals, and the corresponding variance and standard deviation for all the variables included in the analysis. The ICC values for the outcome variables ranged from 0.01 for Sprint to 0.45 for Mental load. The confidence intervals for the ICC values were relatively narrow for most variables, indicating high precision in the estimates. Finally, ICC also suggested statistically significant variability in most of the variables included (ICC > 0.10).

Table 3 shows the differences related to RPE, mental load, TD, and mechanical variables (i.e., ACC, DEC, and HMLD). RPE values were significantly higher on Grass2 compared to Grass1 (*p* < 0.001; ES = 0.38); players covered significantly lower TD on 3G compared to Grass1 (*p* < 0.001; ES = −0.98) and Grass2 (*p* < 0.001; ES = −0.95); and they performed the highest meters per minute on Grass2 compared to Grass1 (*p* < 0.001; ES = 0.36) and 3G (*p* < 0.001; ES = 0.71). Regarding mechanical variables, the ACC number was significantly lower on 3G compared to Grass1 (*p* < 0.001; ES = −0.60) and Grass2 (*p* < 0.001; ES = −0.64); and the DEC number was also significantly lower on 3G compared to Grass 1 (*p* < 0.001; ES. = −0.70) and Grass2 (*p* < 0.001; ES = −0.94). In addition, HMLD was significantly lower on 3G compared to Grass1 (*p* < 0.001; ES = −0.50) and Grass2 (*p* < 0.001; ES = −0.62).

The differences in distances covered at different speed thresholds by playing surface are presented in Table 4. MSR was significantly lower on 3G compared to Grass1 (*p* < 0.05; ES = −0.19) and Grass2 (*p* < 0.001; ES = −0.33); HSR was significantly higher on 3G compared to Grass1 (*p* < 0.05; ES = 0.24); VHSR was significantly higher on Grass2 compared to Grass1 (*p* < 0.001; ES = 0.41) and 3G (*p* < 0.05; ES = 0.18); Sprint was significantly higher on 3G compared to Grass2 (*p* < 0.05; ES = 0.21); and the number of Sprints performed by soccer players were significantly higher on 3G compared to Grass1 (*p* < 0.001; ES = 0.49) and Grass2 (*p* < 0.05; ES = 0.21).

## 4. Discussion

The aim of this study was to examine the influence of playing surface on workload-related variables (i.e., external load, RPE, and mental load) during preseason training sessions in a Spanish professional male soccer team. The main results showed that RPE values, TD, relative TD, ACC, DEC, and HMLD were significantly higher on Grass2, while HSR, Sprint distances, and the number of sprints performed by soccer players were significantly higher on 3G. Even though the playing surfaces were not similar and there was one playing surface of poor quality for practicing professional soccer, to date this is the first study to compare the differences in training load-related variables, including mental load, by different playing surfaces.

Firstly, concerning Mental load, we hypothesized that Mental load values would be greater on artificial turf than on natural turf (Hypothesis 1). Although significant differences were not observed in Mental load, these values were higher on 3G than on both types of grass (i.e., Grass1 and Grass2). These findings could be explained based on previous studies about how players perceive the type of turf in training sessions. When the two types of playing surfaces (i.e., grass vs. artificial turf) are compared, soccer players perceive 3G as harder, more abrasive, and with less grip than grass [47]. Moreover, the general impression of soccer players towards 3G has been previously reported as negative or physically harder to play [32]. The fear of being injured could be another key factor to consider, which could affect perceived mental load [48] since artificial turf has a higher injury risk than grass due to its rigidity and lower shock absorption [49]. 

According to our second hypothesis, we expected that RPE values would be lower on artificial turf than on natural turf (Hypothesis 2). Our results showed higher RPE values on Grass2 compared to the rest of the playing surfaces, similar to some studies that have shown a lower perception of fatigue on artificial turf [33]. The players’ perceptions of the field condition, considering material and quality, could influence their RPE and fatigue during different training sessions. For example, on natural grass, the ball moves faster than other playing surfaces, and training sessions are more demanding. Weather conditions could also affect the surface state, altering training load on both artificial and natural turf, such as drainage quality in different types of fields.

Concerning external load variables, we hypothesized that TD would be higher on artificial turf than on natural turf (Hypothesis 3). The results reported that TD, relative TD, and the number of ACC, DEC, and HMLD were significantly higher on Grass2 compared to the rest of the playing surfaces. Lower values on 3G might be influenced by players’ perceptions that running is more difficult on artificial turf than on natural grass [50]. In addition, the higher quality of natural turf compared to the rest of the playing surfaces could lead to more demanding training sessions, where the ball moves faster. On the contrary, Modric et al. [30] and Brito et al. [31] reported that playing matches on artificial turf resulted in increased running performance during matches. Although these observations belonged to matches, and the players’ characteristics were different (i.e., non-elite young players as opposed to adult male professional players in this study), further research is needed since some studies have not reported clear conclusions about the influence of different types of playing surfaces on external load variables. This is an important issue to be considered in the future, which should also consider internal load variables for getting better player performance during training sessions on different playing surfaces [51].

Research related to the influence of different playing surfaces on training load and match running performance has reported that HSR performance on artificial turf is higher than on natural turf [31,52]. As we hypothesized, our results showed that high-speed variables, such as HSR, Sprint distance, and the number of sprints performed were significantly higher on the 3G than the natural grass (Hypothesis 3), which is likely due to the higher friction and rotational traction achieved on an artificial turf surface providing advantages to the performance of sprints and accelerations [53]. In addition, as Brito et al. [31] hypothesized, natural turf increases fatigue (i.e., higher RPE values), constraining, therefore, the player’s HSR activity. Such a hypothesis may result in more physically demanding training sessions on natural turf with higher values of relative TD, ACC, DEC, and HMLD. However, a study that analyzed the influence of different types of surfaces on a sprint test on young male soccer players indicated that comparing the results of running performance on different surfaces is unjustified, so these results could only provide qualitative information for technical staff [54].

### 4.1. Limitations and Future Directions

It is important to highlight some aspects of the present study to be addressed in future research. Firstly, the playing surfaces were not similar, and there was one playing surface that was not of good quality for practicing professional soccer. Although there are strict guidelines for the quality of soccer turf, there are no such standards for natural surfaces. Consequently, the findings of the present study should be cautiously considered relative to lower-quality surfaces, and further research should address the variability across a range of natural surfaces. Secondly, only one professional soccer team with 20 players was involved in this study, so further studies should include more teams or different club categories (e.g., U21, U18, etc.) to obtain representative results and definitive conclusions on the influence of playing surfaces on training load-related variables. In addition, although the soccer players involved were professional, specific characteristics of the Third Division of Spain could make it difficult to extrapolate the results to other professional contexts. So, future studies should measure how mental load varies between the athletes playing at various competition levels. Likewise, the study results could be influenced by the players’ perceptions of the playing surfaces, which may impact players’ movement or ball behavior. So, further studies could include longitudinal analysis across an entire session to normalize training on different playing surfaces. Finally, there is a lack of studies analyzing the effects of playing surfaces on mental load, so a comparison with previous findings is difficult to make.

### 4.2. Practical Implications

These insights may provide the opportunity for coaches to better understand the influence of playing surfaces in training sessions in professional soccer. Specifically, strength and conditioning coaches should pay special attention to training load management on different playing surfaces, as well as consider different recovery strategies after training sessions to address the demands relative to the playing surface. In addition, they should plan training days according to the pitch surface and its specific demands, considering also the effects of transitions between pitch surfaces that routinely occur during the week [30]. Lastly, although there were no significant differences, the mental load was higher on the 3G surface, so coaches should consider this strategy to increase the mental load demands of training sessions.

## 5. Conclusions

To our knowledge, this is the first attempt, to date, that examines the differences in training workload-related variables considering mental load, RPE, and external load by different playing surfaces with professional male soccer players. The data showed that RPE, TD, relative TD, ACC, DEC, and HMLD were higher on natural grass of high quality, where the ball moved faster than the other playing surfaces, which made training sessions more demanding. However, the data related to distance covered at high speed showed that HSR, Sprint distance, and the number of Sprints performed were higher on 3G, where players could reach higher velocities during training sessions. These findings suggest that playing surface significantly affects the workload in professional male soccer training sessions.

The present study highlights the need for coaches and technical staff to consider the type of training surface in soccer to optimize training load planning and prevent injuries. Training strategies should consider training days and the pitch surface to provide more specific demands. In addition, training on 3G should be necessary to provide stimuli for soccer players to perform greater high-intensity efforts.

## Figures and Tables

**Table 1:** Model information for workload-related variables.

Variables	Marginal R^2^	Conditional R^2^	ICC (CI_95%_)	Variance	SD
RPE	0.03	0.28	0.26 (0.22, 1.29)	0.56	0.75
Mental Load	0.00	0.45	0.45 (0.61, 2.77)	1.31	1.14
TD (m)	0.15	0.19	0.05 (38,084.29, 273,096.20)	115,329.92	339.60
TD_min_ (m·min^−1^)	0.08	0.18	0.11 (7.57, 37.58)	17.52	4.19
ACC (nº.)	0.07	0.25	0.19 (36.68, 161.99)	78.54	8.86
DEC (nº.)	0.13	0.27	0.16 (22.21, 105.68)	50.11	7.08
HMLD (m)	0.06	0.15	0.10 (5806.51, 31,811.13)	14,430.95	120.13

Note. ICC = intraclass correlation coefficient; CI = confidence interval; SD = standard deviation.

**Table 2:** Model information for distances covered-related variables.

Variables	Marginal R^2^	Conditional R^2^	ICC (CI_95%_)	Variance	SD
MSR (m)	0.02	0.11	0.09 (461.04, 2604.87)	1169.88	34.20
HSR (m)	0.01	0.05	0.04 (757.62, 7055.57)	2810.12	53.01
VSHR (m)	0.02	0.12	0.10 (287.77, 1624.74)	727.39	26.97
Sprint (m)	0.01	0.02	0.01 (0.00, 2012.22)	563.95	23.75
Sprints (no.)	0.02	0.12	0.10 (0.73, 4.16)	1.87	1.37

Note. ICC = intraclass correlation coefficient; CI = confidence interval; SD = standard deviation.

**Table 3:** Workload differences by pitch surface.

Variables	Grass1	Grass2	3G	*p*	η^2^	d (CI_95%_)		
	Coeff (SE)	Coeff (SE)	Coeff (SE)			Grass1 vs. Grass2	Grass1 vs. 3G	Grass2 vs. 3G
RPE	5.36 (0.22)	5.93 (0.22)	5.79 (0.29)	a ***	0.03	−0.38 (−0.63, −0.14)	−0.34 (−0.72, 0.04)	0.04 (−0.34, 0.42)
Mental Load	4.99 (0.30)	5.00 (0.30)	5.24 (0.36)		0.00	−0.01 (−0.26, 0.23)	−0.21 (−0.59, 0.16)	−0.20 (−0.58, 0.18)
TD (m)	5279 (137)	5235 (105)	3849 (129)	b ***, c ***	0.15	0.03 (−0.15, 0.20)	0.98 (0.77, 1.19)	0.95 (0.78, 1.13)
TD_min_ (m·min^−1^)	60 (1.36)	64.2 (1.13)	55.2 (1.30)	a ***, b ***, c ***	0.08	−0.36 (−0.54, −0.18)	0.35 (0.14, 0.56)	0.71 (0.53, 0.88)
ACC (nº.)	49.7 (2.5)	50.2 (2.23)	37.5 (2.42)	b ***, c ***	0.07	−0.04 (−0.22, 0.14)	0.60 (0.39, 0.81)	0.64 (0.46, 0.81)
DEC (nº.)	41.3 (2.1)	45.5 (1.82)	28.7 (2.02)	a **, b ***, c ***	0.13	−0.24 (−0.42, −0.06)	0.70 (0.50, 0.91)	0.94 (0.77, 1.12)
HMLD (m)	821 (40.3)	859 (33.2)	622 (38.4)	b ***, c ***	0.06	−0.11 (−0.29, 0.06)	0.50 (0.30, 0.71)	0.62 (0.45, 0.79)

Note. Coeff = coefficient; SE = standard error; m = meters; m·min^−1^ = meters per minute; Grass1 = natural turf of poor quality; Grass2 = natural turf of high quality; 3G = third generation artificial turf; RPE = rating of perceived exertion; TD = total distance covered; ACC = number of accelerations performed at more than 3 m·s^2^; DEC = number of decelerations performed at more than 3 m·s^2^; HMLD = high metabolic load distance; a = significant differences between Grass1 and Grass2; b = significant differences between Grass1 and 3G; c = significant differences between Grass2 and 3G; ** *p* < 0.01; *** *p* < 0.001.

**Table 4:** Differences in distances covered at different speed thresholds by pitch surface.

Variables	Grass1	Grass2	3G	*p*	η^2^	d (CI_95%_)		
	Coeff (SE)	Coeff (SE)	Coeff (SE)			Grass1 vs. Grass2	Grass1 vs. 3G	Grass2 vs. 3G
MSR (m)	176 (11.72)	187 (9.56)	150 (11.15)	b *, c ***	0.02	−0.14 (−0.32, 0.04)	0.19 (−0.01, 0.40)	0.33 (0.16, 0.50)
HSR (m)	164 (23)	192 (17.3)	221 (21.6)	b *	0.01	−0.13 (−0.31, 0.04)	−0.24 (−0.45, −0.04)	−0.11 (−0.28, 0.06)
VSHR (m)	79.9 (8.86)	110.8 (7.36)	95.9 (8.46)	a ***, c *	0.03	−0.41 (−0.59, −0.23)	−0.23 (−0.44, −0.02)	0.18 (0.01, 0.35)
Sprint (m)	83.6 (16.8)	82 (11.3)	125 (15.7)	c *	0.01	0.00 (−0.18, 0.18)	−0.21 (−0.41, 0.00)	−0.21 (−0.38, −0.04)
Sprints (no.)	3.49 (0.46)	4.57 (0.38)	5.48 (0.44)	a **, b ***, c *	0.03	−0.28 (−0.46, −0.10)	−0.49 (−0.70, −0.28)	−0.21 (−0.38, −0.04)

Note. Coeff = coefficient; SE = standard error; m = meters; Grass1 = natural turf of poor quality; Grass2 = natural turf of high quality; 3G = third-generation artificial turf; MSR = medium-speed running distance; HSR = high-speed running distance; VHSR = very-high-speed running distance; Sprint (m) = sprinting speed running distance; Sprints (no.) = number of sprints performed by soccer players; a = significant differences between Grass1 and Grass2; b = significant differences between Grass1 and 3G; c = significant differences between Grass2 and 3G; * *p* < 0.05; ** *p* < 0.01; *** *p* < 0.001.

## Data Availability

The data are available upon request to the corresponding author.

## Supplementary Materials

The following supporting information can be downloaded at: https://www.mdpi.com/article/10.3390/s24144506/s1, Figure S1: Adaptation of the Questionnaire to quantify the Mental Load in Team Sports (QMLST).
