# Supplementary material for: The Influence of the Playing Surface on Workload Response in Spanish Professional Male Soccer Players
Source: Sensors (Basel). 2024 Jul 12;24(14):4506. doi: 10.3390/s24144506 (PMC11281269; doi:10.3390/s24144506)
Supplement: Supplementary file 1 [file sensors-24-04506-s001.zip › sensors-3061326-supplementary.pdf]

	**0**	**1**	**2**	**3**	**4**	**5**	**6**	**7**	**8**	**9**	**10**
**RPE**	Rest	Very, very easy	Easy	Moderate	Somewhat hard	Hard		Very hard			Maximal
**COGNITIVE LOAD**	No effort perceived										Maximun effort perceived

Figure S1. Adaptation of the Questionnaire to quantify the Mental Load in Team Sports (QMLST)
